# Supplementary material for: Comparative genomics provides new insights into the diversity, physiology, and sexuality of the only industrially exploited tremellomycete: Phaffia rhodozyma
Source: BMC Genomics. 2016 Nov 9;17:901. doi: 10.1186/s12864-016-3244-7 (PMC5103461; doi:10.1186/s12864-016-3244-7)
Supplement: Additional file 6: — List of orphan genes with links to PFAM (related to Additional file 1: Table S1). (ZIP 1428 kb) [file 12864_2016_3244_MOESM6_ESM.zip › PFAM_UNIQUE/G02408_P.html]

#                                                                            --- full sequence --- -------------- this domain -------------   hmm coord   ali coord   env coord
  
# target name        accession   tlen query name           accession   qlen   E-value  score  bias   #  of  c-Evalue  i-Evalue  score  bias  from    to  from    to  from    to  acc description of target
  
#------------------- ---------- ----- -------------------- ---------- ----- --------- ------ ----- --- --- --------- --------- ------ ----- ----- ----- ----- ----- ----- ----- ---- ---------------------
  
G02408\_P             -            735 FAM60A               PF15396.1    213       7.7    4.9  10.0   1   2     0.014        20    3.5   1.4   144   168   465   490   430   524 0.51 - Protein Family FAM60A  
G02408\_P             -            735 FAM60A               PF15396.1    213       7.7    4.9  10.0   2   2     0.003       4.5    5.6   1.8   107   161   621   678   553   712 0.55 - Protein Family FAM60A  
G02408\_P             -            735 SOBP                 PF15279.1    306       2.9    6.8  27.9   1   2    0.0022       0.3   10.1  14.6    51   262   313   540   304   554 0.53 - Sine oculis-binding protein  
G02408\_P             -            735 SOBP                 PF15279.1    306       2.9    6.8  27.9   2   2      0.38        52    2.7   4.9   148   254   556   685   538   711 0.43 - Sine oculis-binding protein
